# Serum Uric Acid Is Associated with Left Ventricular Hypertrophy Independent of Serum Parathyroid Hormone in Male Cardiac Patients

**DOI:** 10.1371/journal.pone.0082735

**Published:** 2013-12-10

**Authors:** Shu-ichi Fujita, Yusuke Okamoto, Kensaku Shibata, Hideaki Morita, Takahide Ito, Koichi Sohmiya, Masaaki Hoshiga, Nobukazu Ishizaka

**Affiliations:** Department of Cardiology, Osaka Medical College, Osaka, Japan; University of Alabama at Birmingham, United States of America

## Abstract

**Background:**

Several studies have shown that serum uric acid (UA) is associated with left ventricular (LV) hypertrophy. Serum levels of parathyroid hormone (PTH), which has bbe shown to be correlated with UA, is also known to be associated with cardiac hypertrophy; however, whether the association between UA and cardiac hypertrophy is independent of PTH remains unknown.

**Purpose:**

We investigated whether the relationship between serum uric acid (UA) and LV hypertrophy is independent of intact PTH and other calcium-phosphate metabolism-related factors in cardiac patients.

**Methods and Results:**

In a retrospective study, the association between UA and left ventricular mass index was assessed among 116 male cardiac patients (mean age 65±12 years) who were not taking UA lowering drugs. The median UA value was 5.9 mg/dL. Neither age nor body mass index differed significantly among the UA quartile groups. Patients with higher UA levels were more likely to be taking loop diuretics. UA showed a significant correlation with intact PTH (R = 0.34, P<0.001) but not with other calcium-phosphate metabolism-related factors. Linear regression analysis showed that log-transformed UA showed a significant association with left ventricular mass index, and this relationship was found to be significant exclusively in patients who were not taking loop and/or thiazide diuretics. Multivariate logistic regression analysis showed that log-transformed UA was independently associated with LV hypertrophy with an odds ratio of 2.79 (95% confidence interval 1.48–5.28, P = 0.002 per one standard deviation increase).

**Conclusions:**

Among cardiac patients, serum UA was associated with LV hypertrophy, and this relationship was, at least in part, independent of intact PTH levels, which showed a significant correlation with UA in the same population.

## Introduction

Several previous studies have shown that subjects with higher uric acid (UA) levels more frequently have LV hypertrophy [1], [2], [3]. Serum UA is affected or linked to various factors that may also be associated with LV hypertrophy, including obesity, hypertension, insulin resistance, and chronic kidney disease [4], [5], [6], [7]. The finding that UA level was associated with LV hypertrophy detected by echocardiography independent of renal function, blood pressure, or impaired glucose metabolism [8], [9], although there may be certain gender differences, suggests that the association between UA and LV hypertrophy may not merely be circumstantial.

Parathyroid hormone (PTH), a hormone secreted from parathyroid glands, raises plasma calcium levels by increasing absorption, reducing excretion, and promoting release of calcium from bones; in turn, PTH is regulated by the calcium concentration in the plasma. Besides its effect on calcium homeostasis, serum PTH may have an impact on the development of cardiovascular events [10] and may cause an increase in cardiac mass among the elderly community-dwelling population [11], although studies on whether PTH is associated with cardiac abnormalities, either directly or indirectly via other factors, have not been conclusive [12], [13]. The findings that hyperuricemia and gout occurred with increased frequency among patients with hyperparathyroidism [14] and that serum UA was positively associated with serum PTH in community-dwelling older men [14], [15] suggest that there is a relationship between serum UA and PTH. Such an association may be further supported by the observations that recombinant PTH may induce hyperuricemia [16] and parathyroidectomy reduces serum uric acid levels [17].

Nevertheless, to the best of our knowledge it seems that whether the relationship between serum UA and cardiac hypertrophy, when present, is dependent or independent of serum PTH has not been examined. To this end, we have herein investigated, among cardiac patients, whether the association between UA and LV hypertrophy is dependent on serum PTH levels and other calcium-phosphate metabolism-related parameters, including serum fibroblast growth factor-23 (FGF23), which has also been shown to have a link with cardiac hypertrophy [18], [19].

## Methods

### Ethics

The current retrospective study was approved by the Ethics Committee of Osaka Medical College. The study included only subjects who provided written informed consent for whom sufficient information regarding the data analysis was available.

### Study Population

Between 2012 January and 2012 December, 138 male cardiac inpatients for whom sufficient clinical and echocardiographic information was available were enrolled in the current study. Among the 138 patients, 21 patients and one patient were taking xanthine oxidase inhibitor (allopurinol) and uricosuric drugs, respectively, and were subsequently excluded. As a result, 116 patients, mean age 65±12 years, were included in the current study.

### Laboratory Analysis

Blood samples were collected in the morning after an overnight fast. Aliquots of serum and plasma were obtained and stored at -80 degrees immediately until use. Ca, IP, C-reactive protein (CRP), and B-type natriuretic peptide (BNP) were measured by routine laboratory methods. When serum albumin was 4 mg/dL or lower, serum Ca levels were corrected by using the formula: Ca+(4–“serum albumin”), and designated as corrected Ca (cCa). Serum levels of intact PTH (iPTH) and 25(OH)D were measured using electrochemiluminescence and a competitive protein binding assay (Mitsubishi Medience, Tokyo, Japan). Serum levels of intact FGF23 were measured using a two-step FGF23 enzyme immunoassay (ELISA) kit (Kainos Laboratories Inc., Tokyo, Japan) according to the manufacturer’s instructions. Measurement of FGF23 was performed by two experienced researchers in the institute: the interclass correlation coefficient (ICC) of intra- and inter-operator reliability for FGF23 was 0.98 and 0.95, respectively. The eGFR was calculated by the following Modification of Diet in Renal Disease equation modified for Japanese subjects: eGFR  =  194×(serum creatinine)^−1.094^×(age)^−0.287^ [20]. Among parameters related to calcium-phosphate metabolism, 25(OH)D levels were available for 114 patients (98%).

### Echocardiography

Echocardiographic examinations were performed with a Vivid 7 Dimension equipped with a multi-frequency transducer (GE Healthcare, Vingmed, Norway). Left ventricular end-diastolic dimension (LVDd), interventricular septal thickness (IVST) and posterior wall thickness (PWT) were measured at end diastole. For calculation of the LV mass (LVM), we used the formula proposed by Devereux et al. [21] modified as follows: 0.8×1.04×[(LVDd+IVST+PWT)^3^-LVDd^3^]+0.6 [22]. Body surface area (BSA) was calculated using the following formula: (body weight)^0.425^×(height)^0.725^×0.007184. The LVM index (LVMI) was then calculated as the ratio of LVM to BSA. When the LVMI was greater than 118 g/m^2^, LV hypertrophy was defined to be present [23].

Patients were divided into the following 4 groups according to their LVMI and the relative wall thickness (RWT), which was calculated as PWT×2/LVDd with a partition value of 0.44 [24]: (i) normal geometry, normal RWT and LVMI; (ii) concentric remodeling, increased RWT and normal LVMI; (iii) eccentric hypertrophy, normal RWT and LV hypertrophy; and (iv) concentric hypertrophy, LV hypertrophy and RWT [25].

The LV ejection fraction (LVEF) was calculated either by modified Simpson’s method using the apical 4-chamber view, designated LVEF-Simpson, or by the Teichholz’ formula, designated LVEF-Teichholz [26]. LVEF-Simpson data were available for 103 patients (89%). The ratio of the peak velocity of early filling (E) to the early peak diastolic mitral annulus velocity obtained at the septal and lateral annulus (e’) was measured using pulsed wave tissue Doppler imaging. Among the116 study, patients 92 (79%) had sinus rhythm, 17 (15%) had atrial fibrillation rhythm, and the remaining 7 (6%) had pacemaker rhythm. The value of E/e’, which was used as an index for diastolic LV function for patients with sinus rhythm, was available for 51 patients (55%) of the 92 patients with sinus rhythm.

### Statistical Analysis

Baseline characteristics were assessed with standard descriptive statistics. Data were expressed as either mean ± standard deviation or median and interquartile range. A Spearman rank correlation test was used to assess the correlation between two variables. For multivariate analysis, multivariate linear regression and multivariate logistic regression analyses were used to examine the relationship with, respectively, LVMI and LV hypertrophy. Log-transformed FGF23 (log[FGF23]) and intact PTH (log[iPTH]) were confirmed to be normally distributed by the Kolmogorov-Smirnov test. Data analysis was performed by IBM SPSS statistics version 21.0 (SPSS, Chicago, IL). A value of P<0.05 was taken to be statistically significant.

## Results

### Patient Characteristics

Among the 138 male patients, the median UA level was 5.9 mg/dL. Neither age nor body mass index differed significantly among the four UA quartile groups (Table 1). Ischemic heart disease (IHD) was the most common cardiovascular condition (75/116, 65%). Eighty seven (75%) patients were taking at least one of the following medications: angiotensin converting enzyme inhibitor, angiotensin II receptor blocker, beta adrenergic blocker, or calcium channel blockers; the prevalence of taking medication did not significantly differ among the UA quartile groups. Treatment with loop diuretics was more prevalent in the higher UA quartiles. Twenty four patients (21%) were taking diuretics that may affect serum UA (loop and/or thiazide diuretics); one patient was taking both classes of diuretic. Among 40 patients (34%) diagnosed to have diabetes, 29 were being treated with anti-diabetic medications, and the remaining 11 were diagnosed by their patient history and/or laboratory findings. Because oral glucose challenge test is not included as a routine examination on admission, however, the prevalence of diabetes may have been underestimated.

**Table 1:** Demographic characteristics of the study patients.

	First UA quartile	Second UA quartile	Third UA quartile	Fourth UA quartile	
Variables	(n = 28)	(n = 30)	(n = 29)	(n = 29)	P Value
Uric acid, median (range) mg/dL	4.3 (2.8–4.9 )	5.3 (5.0–5.8)	6.2 (5.9–6.8)	7.6 (6.9–11.0)	<0.001
Age, years	64.4±12.6	66.6±13.3	64.4±11.2	64.7±11.3	0.871
Body mass index, kg/m^2^	23.4±2.9	23.4±2.9	23.4±3.0	23.3±4.1	0.776
Systolic blood pressure, mmHg	129±19	129±20	129±19	125±18	0.646
Pulse rate, bpm	73±14	70±11	73±13	76±25	0.500
Past History					
previous PCI	15 (53.6)	11 (36.7)	14 (48.3)	10(34.5)	0.399
previous CABG	2 (7.1)	4 (13.3)	0 (0.0)	1 (3.4)	0.166
Cardiovascular					
Ischemic heart disease, n (%)	21 (75.0)	19 (63.3)	18 (62.1)	17(58.6)	0.597
Arrhythmic disease, n (%)	6 (21.4)	7 (23.3)	8 (27.6)	4 (13.8)	0.634
Cardiomyopathy, n (%)	2 (7.1)	1 (3.3)	3 (10.3)	1 (3.4)	0.628
NYHA class III/IV, n (%)	1 (3.6)	1 (3.3)	4 (13.8)	6 (20.7)	0.082
Aortic aneurysm, n (%)	1 (3.6)	3 (10.0)	0 (0.0)	3 (10.3)	0.268
Peripheral artery disease, n (%)	1 (3.6)	6 (20.0)	2 (6.9)	1 (3.4)	0.074
Valvular heart disease, n (%)	0 (0.0)	1 (3.3)	4 (13.8)	3 (10.3)	0.148
Cardiac rhythm					
Sinus rhythm, n (%)	22 (78.6)	24 (80.0)	23 (79.3)	23 (79.3)	0.996
Atrial fibrillation, n (%)	5 (17.9)	4 (13.3)	4 (13.8)	4 (13.8)	
Pace maker rhythm, n (%)	1 (3.6)	2 (6.7)	2 (6.9)	2 (6.9)	
Smoking status					
Never, n (%)	5 (17.9)	5 (16.7)	7 (24.1)	4 (13.8)	0.912
Former, n (%)	18 (64.3)	20 (66.7)	15 (51.7)	19 (65.5)	
Current, n (%)	5 (17.9)	5 (16.7)	7 (24.1)	6 (20.7)	
Medication					
ACE inhibitors/ARB, n (%)	14 (50.0)	13 (43.3)	18 (62.1)	18 (62.1)	0.380
Beta blockers, n (%)	7 (25.0)	6 (20.0)	16 (55.2)	14 (48.3)	0.011
Calcium channel blockers, n (%)	10 (35.7)	11 (36.7)	14 (48.3)	13 (44.8)	0.716
Sulfonylurea, n (%)	6 (21.4)	2 (6.7)	3 (10.3)	4 (13.8)	0.387
DPP4 inhibitors, n (%)	3 (10.7)	2 (6.7)	4 (13.8)	1 (3.4)	0.518
Insulin, n (%)	1 (3.6)	3 (10.0)	1 (3.4)	2 (6.9)	0.680
Statin, n (%)	13 (46.4)	16 (53.3)	14 (48.3)	11 (37.9)	0.693
Fibrate, n (%)	0 (0.0)	0 (0.0)	1 (3.4)	0 (0.0)	0.388
Loop diuretics, n (%)	3 (10.7)	1 (3.3)	5 (17.2)	12 (41.4)	0.001
Thiazide diuretics, n (%)	0 (0.0)	0 (0.0)	3 (10.3)	1 (3.4)	0.102
Aldosterone antagonist, n (%)	2 (7.1)	0 (0.0)	2 (6.9)	5 (17.2)	0.102

UA, uric acid; PCI, percutaneous coronary intervention; CABG, coronary artery bypass surgery; ACE, angiotensin converting enzyme; ARB, angiotensin II receptor blockers; DPP4, dipeptidyl peptidase-4.

### Serum UA and Laboratory and Echocardiographic Parameters

Laboratory and echocardiographic data were compared across the UA quartile groups by the Kruskal-Wallis test (Table 2). Serum creatinine increased in accordance with the UA value. Among the parameters related to calcium-phosphate metabolism, intact PTH was highest among patients in the top UA quartiles. LVMI was higher in the third and fourth UA quartile; on the other hand, RWT did not significantly differ among the groups (Table 2). The correlation coefficient between LVEF-Simpson and LVEF-Teichholz was 0.564 (P<0.001). Either LVEF-Simpson or LVEF-Teichholz differed significantly between the groups. LVEF-Teichholz was significantly smaller in the highest UA quartile than in the lower three UA quartiles (P = 0.029). We did not regard this as a true difference, however, because (i) such a relationship was not observed when LVEF-Simpson was used instead, and (ii) LVEF determined by the Teichholz’ formula may be less accurate when applied to a population that includes subjects who have abnormal regional LV wall motion, such as the current study population. E/e’ in the patients with sinus rhythm was not different among UA quartile groups.

**Table 2:** Laboratory and echocardiographic data.

	First UA quartile	Second UA quartile	Third UA quartile	Fourth UA quartile	
Variables	(n = 28)	(n = 30)	(n = 29)	(n = 29)	P Value
White blood cell count, x10^3^/µL	5.58(4.49–6.58)	5.51 (4.98–6.78)	6.27 (4.89–7.15)	6.12 (4.95–7.41)	0.440
Hemoglobin, g/dL	14.3 (12.9–15.0)	13.8 (12.8–14.9)	14.5 (13.2–15.3)	13.4 (12.2–14.4)	0.103
Platelet count, x10^4^/µL	19.7 (16.8–22.5)	22.3 (17.0–25.5)	21.0 (16.6–25.3)	21.4 (19.2–25.1)	0.563
Total protein, g/dL	6.8 (6.5–7.3)	7.1 (6.9–7.3)	6.8 (6.4–7.2)	6.9 (6.8–7.2)	0.146
Albumin, g/dL	4.1 (3.8–4.3)	4.1 (3.8–4.3)	3.9 (3.7–4.2)	3.9 (3.7–4.1)	0.301
Alanine aminotransferase, IU/L	19.5 (16.0–29.5)	24.0 (13.8–43.0)	20.0 (15.0–28.5)	18.0 (15.0–30.0)	0.764
Blood urea nitrogen, mg/dL	16 (14–18)	16 (13–17)	16 (12–19)	18 (14–22)	0.103
Serum creatinine, mg/dL	0.82 (0.69–0.95)	0.86 (0.75–0.97)	0.90 (0.78–1.03)	0.98 (0.82–1.27)	0.013
eGFR, mL/min/1.73 m^2^	52.1 (43.4–66.6)	50.3 (42.7–63.7)	47.8 (41.2–56.3)	48.0 (32.8–54.4)	0.078
C-reactive protein, mg/dL	0.08 (0.02–0.15)	0.12 (0.03–0.40)	0.05 (0.03–0.23)	0.07 (0.04–0.47)	0.547
B-type natriuretic peptide, pg/mL	23.4 (10.3–74.6)	27.6 (14.6–55.0)	22.7 (9.9–51.3)	66.9 (18.7–231)	0.120
Corrected calcium, mg/dL	9.2 (8.8–9.3)	9.0 (8.8–9.3)	9.0 (8.8–9.2)	9.2 (9.1–9.4)	0.183
Inorganic phosphate, mg/dL	3.2 (2.8–3.6)	3.3 (2.9–3.8)	3.3 (2.9–3.7)	3.4 (3.0–3.8)	0.602
	31.0(24.3–42.0)	36.5 (30.0–43.3)	(24.5–48.5)	33.5–59.0)	
FGF23, pg/mL	49.7 (33.5–80.1)	71.9 (48.0–136)	54.7 (28.8–73.9)	66.6 (45.1–86)	0.097
25(OH) vitamin D, pg/mL (n = 114)	24.9 (17.1–31.9)	19.4 (16.2–23)	20.3 (16.0–25.3)	20.1 (18.3–25)	0.152
Echocardiographic data					
LVDd, mm	4.9 (4.5–5.3)	4.8 (4.5–5.3)	4.8 (4.7–5.3)	5.4 (4.9–6.1)	0.036
LVDs, mm	3.1 (2.9–3.6)	3.0 (2.7–3.5)	3.2 (2.9–3.7)	3.5 (3.0–4.7)	0.054
IVST, mm	0.9 (0.9–1.0)	1.0 (0.9–1.1)	1.0 (0.9–1.1)	1.0 (0.9–1.1)	0.267
PWT, mm	1.0 (0.9–1.0)	1.0 (0.9–1.1)	1.0 (0.9–1.1)	1.0 (0.8–1.1)	0.523
LV mass index, g/m^2^	100 (83–109)	98 (81–124)	100 (88–116)	123 (92–154)	0.022
Relative wall thickness	0.41 (0.35–0.43)	0.39 (0.35–0.48)	0.41 (0.36–0.44)	0.37 (0.30–0.45)	0.544
LVEF-Simpson, % (n = 103)	59 (51–64)	62 (54–65)	60 (56–64)	60 (41–66)	0.870
LVEF-Teichholz, %	65 (56–70)	63 (59–73)	62 (58–69)	59 (44–68)	0.176
Left atrial dimension, mm	4.0 (3.2–4.5)	3.8 (3.3–4.1)	3.9 (3.6–4.5)	4.0 (3.6–4.5)	0.400
E/e' (n = 51)	8.9 (6.4–10.3)	7.8 (6.2–10.4)	8.1 (6.5–10.7)	10.8 (8.0–13.0)	0.314
Left ventricular geometry					
Normal	20 (71.4)	15 (50.0)	19 (65.5)	9 (31.0)	0.022
Concentric remodeling, n (%)	5 (17.9)	6 (20.0)	3 (10.3)	3 (10.3)	
Eccentric hypertrophy, n (%)	3 (10.7)	5 (16.7)	4 (13.8)	12 (41.4)	
Concentric hypertrophy, n (%)	0 (0.0)	4 (13.3)	3 (10.3)	5 (17.2)	

LVDd, left ventricular end-diastolic dimension; LVDs, left ventricular end-systolic dimension; IVST, interventricular septal thickness; PWT, posterior wall thickness; LVEF, left ventricular ejection fraction.

By Spearman’s rank correlation test, serum creatinine, intact PTH, BNP, and LVMI were positively, and eGFR was negatively, associated with serum UA (Table 3). LVEF-Teichholz, but not LVEF-Simpson, showed a significant correlation with serum UA. Serum UA was correlated significantly with LVMI in 75 patients with ischemic heart disease (R = 0.261, P = 0.024) and in 41 patients without (R = 0.327, P = 0.037). Serum UA was correlated with LVMI significantly in 40 patients with diabetes (R = 0.398, P = 0.011) and in 76 patients without diabetes (R = 0.253, P = 0.028).

**Table 3:** Spearman’s correlation coefficients for the association with serum UA.

	R	P value
Age	–0.13	0.169
Body mass index	–0.02	0.854
Systolic blood pressure	–0.01	0.896
Serum creatinine	0.27	0.003
eGFR	–0.20	0.028
B-type natriuretic peptide	0.18	0.047
Corrected calcium	0.04	0.680
Inorganic phosphate	0.09	0.335
Intact parathyroid hormone	0.34	<0.001
FGF23	0.07	0.482
25(OH) vitamin D, pg/mL (n = 114)	–0.10	0.298
LV mass index, g/m^2^	0.30	0.001
Relative wall thickness	–0.16	0.092
LVEF-Simpson, % (n = 103)	–0.03	0.752
LVEF-Teichholz, %	–0.21	0.022
Left atrial dimension, mm	0.14	0.130
E/e' (n = 51)	0.15	0.279

LVEF, left ventricular ejection fraction.

### Multivariate Analysis

Multivariate linear regression model showed that serum UA was significantly associated with LVMI after adjustment for age (Table 4, model 1), and this association remained significant after further adjustment for blood pressure and eGFR (model 2), and serum calcium-phosphate metabolism-related parameters (cCa, IP, intact PTH, and FGF23) (model 3). The strength of the association was not attenuated when model 3 was applied to patients who were not taking either loop or thiazide diuretics; however, this model was not significant (P>0.05) when applied to patients taking these diuretics. We did not include 25(OH)D in the model because it did not have a significant correlation with UA (Table 3) or with LVMI by univariate analysis (standardized β = 0.071, P = 0.455).

**Table 4:** Multivariate linear regression analysis examining the association between various parameters and left ventricular mass index.

	Model 1			Model 2			Model 3			Diuretics (-)			Diuretics (+)		
	R^2^ = 0.146			R^2^ = 0.167			R^2^ = 0.278			R^2^ = 0.333					
Predictors	Std β		P value	Std β		P value	Std β		P value	Std β		P value	Std β		P value
log(UA)	0.35		<0.001	0.35		<0.001	0.24		0.011	0.28		0.006	–0.07		0.817
Age	0.21		0.018	0.18		0.072	0.16		0.083	0.14		0.156	0.45		0.202
Systolic blood pressure	-		-	0.15		0.098	0.18		0.039	0.32		0.001	0.18		0.468
eGFR	-		-	0.01		0.906	0.06		0.554	0.08		0.445	0.48		0.096
log(cCa)	-		-	-		-	–0.03		0.713	0.08		0.411	–0.26		0.224
log(IP)	-		-	-		-	0.19		0.031	0.23		0.020	0.29		0.238
log(iPTH)	-		-	-		-	0.25		0.008	0.23		0.024	0.16		0.440
log(FGF23)	-		-	-		-	0.14		0.114	–0.01		0.954	0.48		0.061

β, the standardized correlation coefficient. Diuretics indicate medication with loop and/or thiazide diuretics. Std

**Figure 1:**
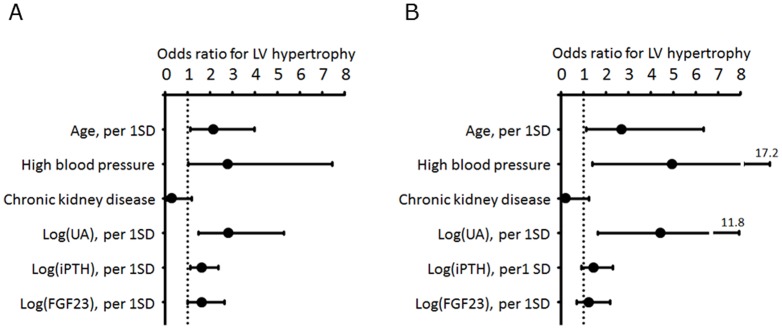
Multivariate logistic regression analysis using left ventricular (LV) hypertrophy as the independent variable. Odds ratios for age, log-transformed uric acid (log[UA]), intact parathyroid hormone (log[iPTH]), and FGF23 (log[FGF23]) were calculated on the basis of an increase in a one standard deviation (SD) for each variable. A. Whole study population (n = 116). B. Patients who were not taking loop and/or thiazide diuretics (n =  92). High blood pressure indicates systolic blood pressure≥140 mmHg and/or diastolic blood pressure≥90 mmHg, and chronic kidney disease indicates eGFR<60 mL/min.1.73 m^2^.

Next, we analyzed the association of age, high blood pressure (systolic blood pressure≥140 mmHg and/or diastolic blood pressure≥90 mmHg), chronic kidney disease (eGFR<60 mL/min.1.73 m^2^), UA, intact PTH, and FGF23 with LV hypertrophy by multivariate logistic regression analysis. We included FGF23 in this model because we previously found a significant association between FGF23 and LVMI in cardiac patients [19]. Log(UA) was found to be significantly associated with LV hypertrophy with an odds ratio of 2.79 (95% CI 1.47-5.28, per 1 SD increase) in the whole study population, and with an odds ratio of 4.42 (95% CI 1.66-11.77, per 1 SD increase) in patients who were not taking either loop or thiazide diuretics (Figure 1). Next, eccentric LV hypertrophy or concentric LV hypertrophy was used as a dependent variable in place of LVMI in a model using the same independent variables. Log(UA) was significantly associated with eccentric LV hypertrophy with an odds ratio of 2.51 (95% CI 1.27-4.94, per 1 SD increase, P = 0.008), but in this model, the association between log(UA) and concentric LV hypertrophy did not reach statistical significance (odds ratio 1.72 [95% CI 0.74-4.01], per 1 SD increase, P = 0.206).

## Discussion

In the current study, we investigated whether the relationship between serum UA and LV hypertrophy, if present at all, is independent of serum intact PTH, which may be associated with serum UA. In fact, intact PTH was significantly correlated with UA with a correlation coefficient of 0.335 (P<0.001). Both UA and intact PTH were associated with LV hypertrophy by univariate analysis. The association between serum UA and LVMI, and that between serum UA and LV hypertrophy were shown to be independent of serum intact PTH and other possible confounders, including age, blood pressure, renal function (eGFR), by linear regression and logistic regression analyses, respectively.

To date, several studies have investigated the association between serum UA and LV hypertrophy. It is also important to determine whether the observed association, if present, is dependent or independent of other possible confounding variables because higher serum UA levels may be associated with hypertension, renal dysfunction, diabetes, conditions which are also related to LV hypertrophy [27], [28]. For example, in an analysis of 3305 essentially healthy male individuals, Mitsuhashi et al. showed that individuals with UA values of 6.6–11.0 mg/dL had an increased prevalence of LV hypertrophy, which was independent of age, body mass index, serum creatinine, hypertension, diabetes and hyperlipidemia [2]. In that study, unlike in the current one, electrocardiographic criteria were used for the determination of LV hypertrophy. In addition, Viazzi et al. demonstrated that the association between serum UA and cardiac hypertrophy remained significant after adjustment for body mass index, age, creatinine clearance, and high-density lipoprotein cholesterol in middle-aged untreated female patients with essential hypertension [8]. In the Framingham Offspring Cohort, Krishnan et al. also found a significant association between serum UA and LV wall thickening, however, the observed association lost statistical significance after multivariate adjustment [1]. Little seems to be known about the mechanism, if there is one, by which UA induces myocardial hypertrophy; possibilities may include activation of the renin-angiotensin system, enhanced production of reactive oxygen species, and upregulation of endothlin-1 expression [29], [30].

Similar to the case of serum UA, the relationship between PTH and LV hypertrophy has been examined in several previous studies. Fujii et al. reported that a relationship between intact PTH and LV hypertrophy in patients with chronic dialysis may be observed only when intact PTH levels are extremely high [12]. On the other hand, higher PTH concentrations were associated with greater LV mass in an older-aged community-dwelling cohort [11], and plasma PTH was an independent predictor of LV hypertrophy in patients undergoing chronic hemodialysis [31]. We also found that serum intact PTH was positively associated with LV hypertrophy among cardiac patients [19]. PTH may induce hypertrophy of cardiomyocytes via activating MAPK pathway [32].

Several studies have described the association between serum UA and PTH. Serum UA levels were significantly correlated with intact PTH levels among patients with primary hyperparathyroidism [33]. In addition, serum UA levels were associated with serum calcium, PTH, and 25(OH)D after adjusting for potential confounders among community-dwelling elderly men [15]. The finding that parathyroidectomy reduced serum UA [34] may be explained by the fact that PTH modulates serum UA mainly through changes in uric acid clearance [35]. On the other hand, to the best of our knowledge, no studies have examined whether the relationship observed between serum UA and LV hypertrophy is dependent or independent of PTH.

In the current study, we showed that serum UA was associated with LV hypertrophy independently of PTH levels and other possible confounders, including age, high blood pressure chronic kidney disease, and FGF23 level, in cardiac patients. In this study population, PTH also showed a positive and independent association with LV mass. The observed relationship observed between UA and LV hypertrophy is not likely to be due to diuretic medications, which can affect serum UA levels, for patients with cardiac hypertrophy and heart failure, because the association between serum UA and LV hypertrophy was observed among patients who were not taking loop and/or thiazide diuretics. Due to the small number of the subjects who were taking aldosterone antagonists, we did not assess whether such drugs would modify the relationship between serum UA and LV hypertrophy. Along with the measurement of renin or aldosterone concentrations, this point should be examined in future studies, as it was recently suggested that activation of mineralocorticoid receptor and secretion of PTH may mutually affected [36].

It was found that serum UA was significantly associated with eccentric, but no concentric, LV hypertrophy; however, these assessments should be repeated among a higher number of study patients. We also found that serum UA was not associated with LVEF determined by modified Simpson’s method. Whether patients with hitgher serum UA are more susceptible to LV hypertrophy than systolic dysfunction awaits evaluation in future studies.

Several attempts have recently been made to evaluate whether reduction of UA will lead to an improvement in cardiac abnormalities. For example, Rekharaj et al. demonstrated that allopurinol regressed LV hypertrophy and improved endothelial function in patients with ischemic heart disease [37], and Szwejkowski et al. showed that allopurinol regressed LV mass in diabetic patients [38]. Our findings provide additional information on whether or not modulation of serum UA will lead to the amelioration of cardiac geometrical abnormalities.

There are several limitations in the current study. First, we enrolled cardiac inpatients with a wide variety of cardiac abnormalities; therefore, whether UA represents an independent risk factor for LV hypertrophy in patients with a specific condition―hypertrophic cardiomyopathy, for example―requires further investigation. Second, we excluded patients who were taking UA lowering agents. Whether the association between serum UA and LV hypertrophy might be modified by certain UA lowering drugs should be assessed in future studies.

In summary, among male cardiac patients, serum UA was associated with LV hypertrophy independent of intact PTH levels, which showed a significant correlation with serum UA, and other possible confounders, including blood pressure and renal function. Whether strategies leading to UA lowering would improve cardiac performance by decreasing LV mass in cardiology patients awaits further investigation.
